# Determinants of exercise capacity in cystic fibrosis patients with mild-to-moderate lung disease

**DOI:** 10.1186/1471-2466-14-74

**Published:** 2014-04-30

**Authors:** Jean Pastré, Anne Prévotat, Catherine Tardif, Carole Langlois, Alain Duhamel, Benoit Wallaert

**Affiliations:** 1 Université Lille 2 et Clinique des Maladies Respiratoires, CRCM Hôpital Calmette, CHRU Lille, France; 2 Service de Physiologie Respiratoire, CHRU Rouen, France; 3 Unité de Biostatistiques, CHRU Lille, France

**Keywords:** Cystic fibrosis, Cardiopulmonary exercise testing, Pulmonary function, Exercise

## Abstract

**Background:**

Adult patients with cystic fibrosis (CF) frequently have reduced exercise tolerance, which is multifactorial but mainly due to bronchial obstruction. The aim of this retrospective analysis was to determine the mechanisms responsible for exercise intolerance in patients with mild-to-moderate or severe disease.

**Methods:**

Cardiopulmonary exercise testing with blood gas analysis at peak exercise was performed in 102 patients aged 28 ± 11 years: 48 patients had severe lung disease (FEV_1_ < 50%, group 1) and 54 had mild-to-moderate lung disease (FEV_1_ ≥ 50%, group 2). VO_2_ peak was measured and correlated with clinical, biological, and functional parameters.

**Results:**

VO_2_ peak for all patients was 25 ± 9 mL/kg/min (65 ± 21% of the predicted value) and was < 84% of predicted in 82% of patients (100% of group 1, 65% of group 2). VO_2_ peak was correlated with body mass index, C-reactive protein, FEV_1_, FVC, RV, DLCO, V_E_/VCO_2_ peak, V_D_/V_T_, PaO_2_, PaCO_2_, P(A-a)O_2_, and breathing reserve. In multivariate analysis, FEV_1_ and overall hyperventilation during exercise were independent determinants of exercise capacity (R^2^ = 0.67). FEV_1_ was the major significant predictor of VO_2_ peak impairment in group 1, accounting for 31% of VO_2_ peak alteration, whereas excessive overall hyperventilation (reduced or absent breathing reserve and V_E_/VCO_2_) accounted for 41% of VO_2_ alteration in group 2.

**Conclusion:**

Exercise limitation in adult patients with CF is largely dependent on FEV_1_ in patients with severe lung disease and on the magnitude of the ventilatory response to exercise in patients with mild-to-moderate lung disease.

## Background

Cystic fibrosis (CF) is characterized by deterioration of nutritional status and irreversible loss of lung function [1-3]. Patients with CF often experience exertional dyspnea and have reduced maximal exercise capacity, which is an important predictor of mortality [4-7]. Regular exercise in these patients has been associated with improved aerobic exercise endurance and quality of life [4,8]. Physical exercise requires the cardiopulmonary system to deliver oxygen to muscles in sufficient quantity to generate energy through aerobic glycolysis. There are conflicting data on the precise mechanisms underlying exercise intolerance in CF, and a number of factors have been implicated [9], including poor nutritional status, peripheral muscle dysfunction [10,11], and especially, ventilatory limitation [12,13]. In other studies, dysfunctional gas exchange has been shown to play a crucial role in limiting exercise performance [14-17].

Only a third of the variability in exercise capacity of CF patients can be explained by FEV_1_, demonstrating that resting pulmonary function tests (PFTs) alone are insufficient to explain the exercise limitation [1,9,13]. By comparison, cardiopulmonary exercise testing (CPET) offers a sensitive evaluation of potential physiological disturbances in cardiovascular, respiratory, peripheral, or neurosensory responses to a standardized exercise protocol [18]. Although it remains underutilized in CF [19], CPET could provide important exercise-related measures that might explain the reduced exercise performance and thus assist in CF patient management aimed at improving exercise capacity.

With this in mind, we initiated a study to determine the mechanisms responsible for exercise limitation in 102 adult CF patients with mild-to moderate or severe lung disease. The patients were subjected to CPET with blood gas analysis during exercise and the results were correlated with clinical and functional characteristics.

## Methods

### Patients

A total of 102 adult patients (sex ratio M:F 0.52) with CF were enrolled at four CF centers in France: Lille (75 patients), Rouen (15 patients), Dijon (5 patients), and Grenoble (7 patients)*.* Written informed consent for participation in the study was obtained from participants. Use of the patient data was approved by the local ethics committee, and the study was considered observational and approved as such by the Institutional Review Board of the French Learned Society for Pulmonology (*Société de Pneumologie de Langue Française*, CEPRO 2012 009).

Clinical, nutritional, biological, PFT, and CPET data were obtained on the same day, either at diagnosis or at the routine annual evaluation, and were retrospectively collected. When patients were seen at several follow-up visits, only the data from the first visit were recorded. Hypoxemic patients did not perform CPET and were excluded from the analysis. A diagnosis of CF was obtained by sweat chloride > 60 mmol/L and the presence of CFTR gene mutations by molecular analysis. Additional characteristics recorded at the time of testing were diseases usually associated with CF, bacterial colonization, treatments, and nutritional status, including height and weight measurements and impedance analysis.

### Cardiopulmonary testing

Forced vital capacity (FVC), forced expiratory volume in 1 s (FEV_1_), FEV_1_ to FVC ratio, total lung capacity (TLC), and residual volume (RV) were measured by plethysmography (Jaeger-Masterlab® cabin). Diffusing capacity of the lung for carbon monoxide (DLCO: mL CO/min/mm Hg) was adjusted for hemoglobin concentration in g/dL according to Cotes’ equation: corrected (Hb) DLCO = DLCO × (10.2 + Hb)/(1.7 × Hb). Following ATS/ERS 2005 guidelines, the lower limits of normal were set at the 5th percentile (or predicted minus 1.64 SD) of each reference population. The results are expressed as percentages of the predicted values. Predicted normal values were derived from standard equations [20-22].

PFTs and CPET were performed in an air-conditioned laboratory (22°C constant temperature), using a standardized protocol as previously described [23,24]. The CPET protocol was the same at each center. Each patient underwent a symptom-limited incremental exercise test on an ergometric bicycle (Ergoline-Ergometrics 800®). The protocol included a warm-up period of 3 min at 20 W followed by a progressively increasing work rate (WR) in a ramp fashion and then 3 min recovery. The ramped WR increment was individualized (range, 8–30 W/min). During exercise, heart rate (HR) was monitored continuously by 12-lead ECG, and arterial oxygen saturation (SpO_2_) was measured by pulse oximetry (Nellcor N-395). The expired gases were analyzed with an Ergocard®, focusing on oxygen consumption (VO_2_), carbon dioxide production (VCO_2_), minute ventilation (VE), and tidal volume (VT). The oxygen pulse (VO_2_/HR) was calculated. Measurements of PaO_2_ and PaCO_2_ were performed on room air at rest and at peak exercise. Normal values for PaO_2_ were derived from [25]. Lactatemia was determined at maximal exercise. Breathing reserve (BR) was calculated as BR = (predicted maximum minute ventilation [MMV] – V_E_ peak)/MMV, with MMV estimated from MMV = FEV_1_ × 40. HR peak was expressed as a percentage of maximum predicted HR, calculated as HR max = 210 – (0.65 × age). Dead space (V_D_/V_T_) was calculated according to Bohr’s equation corrected for the additional instrument dead space: V_D_/V_T_ = (PaCO_2_ – PECO_2_ mean)/PaCO_2_ – (V_D_ [machine]/V_T_) where PECO_2_ is the partial pressure of expired CO_2_. Predicted values for VO_2_ max were calculated from reference equations [26]. Poor motivation appeared not to be an interfering factor in our analysis, as all patients had at least one of the following: BR < 15%, peak HR > 90% of predicted, peak lactate > 7 mmol/L, peak exercise PaO_2_ < 55 mm Hg, and peak V_E_/VO_2_ > 35 or peak RER > 1.15 [23]. Immediately after exercise, subjects were asked to score their sense of breathlessness and muscle fatigue at peak exercise using Borg scales.

### Statistical analysis

The continuous variables are reported as mean ± SD. Normal distribution of quantitative variables was tested by the Shapiro–Wilk test. Differences in FEV_1_ between the groups were determined with the Student’s t-test or Mann–Whitney test. Bivariate analyses were performed to study the relationships between each explanatory variable and the VO2 peak. Pearson’s or Spearman’s correlation coefficient was used for quantitative variables, and the Student’s t-test or Mann–Whitney test for qualitative variables. In addition, a multivariable linear regression with a stepwise selection at the level 0.2 was performed to identify a subset of the most important explanatory variables for the relationship with VO2 peak. In order to avoid the problem of multicolinearity which happens when the explanatory variables are highly correlated, and to obtain a parsimonious model, we adopt the following strategy: first, variables with p < 0.2 were selected and included in a Principal Component Analysis (PCA) in order to study their correlations. Then, the variables included in the multivariable regression model were selected by the results of PCA (graphic correlation circle) on the basis of their clinical pertinence. The stability of the model was assessed by a bootstrap method [27]. The bootstrap resampling method was based on 1000 replicates of the initial dataset. Multivariable regression with a stepwise selection at the level 0.2 was performed on each of these replicates. The inclusion of the variable in the final model was confirmed if this candidate variable was selected in at least 70% of these 1000 analyses. In the final model, for each variable we computed the partial R-square, coefficient, 95% confidence intervals and adjusted p-value. Final variables from the multivariate analysis were applied to each group of FEV_1_. All analyses were achieved with SAS software version 9.2 (SAS Institute Inc., Cary, NC). All tests were performed at the significant level at 0.05.

## Results

### Subjects

The demographic and clinical characteristics are shown in Table 1, and the resting PFT results are presented in Table 2. The cohort consisted of 102 CF patients with a mean age of 28 ± 11 years (range 17–67). The time from diagnosis to evaluation was 16 ± 10 years. For data analysis, the cohort was divided into two groups according to their FEV_1_: group 1 patients with severe lung disease (FEV_1_ < 50%, 48 patients) and group 2 patients with mild-to-moderate lung disease (FEV_1_ ≥ 50%, 54 patients). Group 1 had a significantly higher frequency of homozygosity for the CFTR ∆F 508 mutation, pancreatic insufficiency, bronchial colonization with *Pseudomonas aeruginosa*, and biological inflammatory syndrome, and significantly lower body mass index (BMI) and longer disease duration (data not shown).

**Table 1:** Demographic and clinical characteristics of the CF patients


∆F 508 homozygous mutation^a^	31/79 (40%)
Smoker^a^	10/102 (10%)
Oxygen supplementation^a^	11/95 (11%)
ABPA^a^	33/95 (35%)
Exocrine pancreatic insufficiency^a^	78/102 (76%)
Diabetes^a^	21/102 (21%)
Nasal polyposis^a^	25/94 (27%)
BMI, kg/m^2b^	20 (20 ± 3)
Lean body mass, kg^b^	45 (48 ± 9)
*Pseudomonas aeruginosa*^a^	63/99 (63%)
*Staphylococcus aureus* MS^a^	36/99 (36%)
*Mycobacterium* abscess^a^	4/99 (4%)
Blood leukocytes, 10^12^/mm^3b^	9 (9.7 ± 3.8)
Blood polymorphonuclear neutrophils, 10^12^/mm^3b^	6 (6.4 ± 3.3)
CRP, mg/L^b^	5.5 (15 ± 30)
Serum albumin, g/L^b^	42 (42 ± 4)

ABPA = allergic bronchopulmonary aspergillosis; BMI = body mass index; CRP = C-reactive protein.

Qualitative variables are given as frequency and percentage.

^a^Results are expressed as number (percentage) of patients.

^b^Results are expressed as median values (mean ± SD).

**Table 2:** **Resting pulmonary function tests in CF patients classified according to FEV**_**1**_

	**All patients (n = 102)**	**Group 1 (n = 48)**	**Group 2 (n = 54)**
FEV_1_		< 50%	≥ 50%
FEV_1_^a^	60 ± 28	35 ± 9^b^	82 ± 18
FVC^a^	75 ± 24	56 ± 14^b^	93 ± 16
FEV_1_/FVC	65 ± 15	54 ± 11^b^	75 ± 10
RV^a^	176 ± 65	220 ± 50^b^	135 ± 48
DLCO^a^	68 ± 18	56 ± 13^b^	78 ± 14
PaO_2_, mm Hg	80 ± 14	71 ± 10^b^	87 ± 12
PaCO_2_, mm Hg	38 ± 4	38 ± 5	37 ± 4
P(A-a)O_2_, mm Hg	29 ± 13	38 ± 8^b^	21 ± 12

^a^Results are expressed as mean ± SD percentage of predicted values.

^b^*P* < .0001 compared with group 2.

The patients had a range of disease severities and were recruited from four centers in France. Patients from different centers all had characteristics consistent with the French CF Registry 2009 Report [28] and showed equivalent frequencies of key CF characteristics (∆F508 mutation, exocrine pancreatic insufficiency, and colonization with *P. aeruginosa*), supporting the reproducibility of the results and the potential for extrapolation to other patient populations.

Resting PFT values (Table 2) were significantly more altered in group 1 than in group 2 (Table 2). As expected, three major functional abnormalities were found: obstructive syndrome (FEV_1_/FVC = 65 ± 15% of predicted), altered distension (RV = 176 ± 65% of predicted), and altered DLCO (68 ± 18% of predicted).

### Exercise responses

Exercise cessation was mainly due to leg fatigue in combination with dyspnea (62%), whereas leg fatigue alone or dyspnea alone was observed in 17% and 21% of patients, respectively. The VO_2_ peak value (weight-adjusted VO_2_) was decreased to < 84% of predicted in 83/102 (82%) of patients (48/48 [100%] in group 1 and 35/54 [65%] in group 2) and was significantly lower in group 1 than in group 2 (Table 3).

**Table 3:** **Cardiopulmonary exercise tests in CF patients classified according to FEV**_**1**_

	**All patients (n = 102)**	**Group 1 (n = 48)**	**Group 2 (n = 54)**
FEV_1_		< 50%	≥ 50%
VO_2_ peak, mL/kg/min	25 ± 9	20 ± 5^d^	30 ± 8.5
VO_2_ peak^a^	65 ± 21	51 ± 13^d^	77 ± 20
Borg dyspnea	4.7 ± 1.9	5.6 ± 2^b^	4.1 ± 1.5
Borg Leg fatigue	4.5 ± 1.8	5.2 ± 1.8^b^	4.4 ± 1.7
VE peak, L/min	58 ± 22	44 ± 11^d^	70 ± 21
RR peak, min	41 ± 9	43 ± 9^b^	39 ± 8
V_T_/FVC peak, %	48 ± 11	47 ± 13	48.6 ± 8
V_E_/VO_2_ peak	41 ± 7	42 ± 7	41 ± 7
V_E_/VCO_2_ peak	36 ± 6	37 ± 6	35 ± 5
BR, %	24 ± 20	8 ± 11^d^	37.5 ± 16
V_D_/V_T_ peak	0.32 ± 0.11	0.39 ± 0.07^d^	0.25 ± 0.10
pH peak	7.34 ± 0.04	7.34 ± 0.04	7.35 ± 0.04
PaO_2_ peak, mm Hg	76 ± 16	63 ± 10^d^	89 ± 12
PaCO_2_ peak, mm Hg	40 ± 7	44 ± 6^d^	36 ± 4
P(A-a)O_2_ peak, mm Hg	37 ± 13	46 ± 8^d^	28 ± 10
Lactatemia peak, mmol/L	7 ± 2.6	6 ± 2^d^	7.9 ± 3
HR peak^a^	82 ± 10	79 ± 8^d^	86 ± 9
VO_2_/HR peak^a^	79 ± 22	67 ± 16^d^	90 ± 21

^a^Results are expressed as mean ± SD percentage of predicted values.

^b^*P* < .01 compared with group 2.

^c^*P* < .001 compared with group 2.

^d^*P* < .0001 compared with group 2.

Analysis of the ventilatory response (V_E_ peak, BR, respiratory rate (RR), V_T_/FVC peak) highlighted the differences according to FEV_1_ impairment (Table 3). Group 1 had a lower absolute value of V_E_ at peak exercise, and a depletion of BR. Hyperventilation was due to simultaneous increases in RR and tidal volume. Impairment in pulmonary gas exchange was more severe in group 1, as shown by higher values of P(A-a)O_2_, V_D_/V_T_ peak, and PaCO_2_, and lower values of PaO_2_. Cardiocirculatory responses were normal in group 2, but patients in group 1 showed low VO_2_/HR values and a significant decrease in peak HR. Four patients experienced ECG abnormalities but continued with the exercise test.

### Determinants of exercise capacity

**Figure 1:**
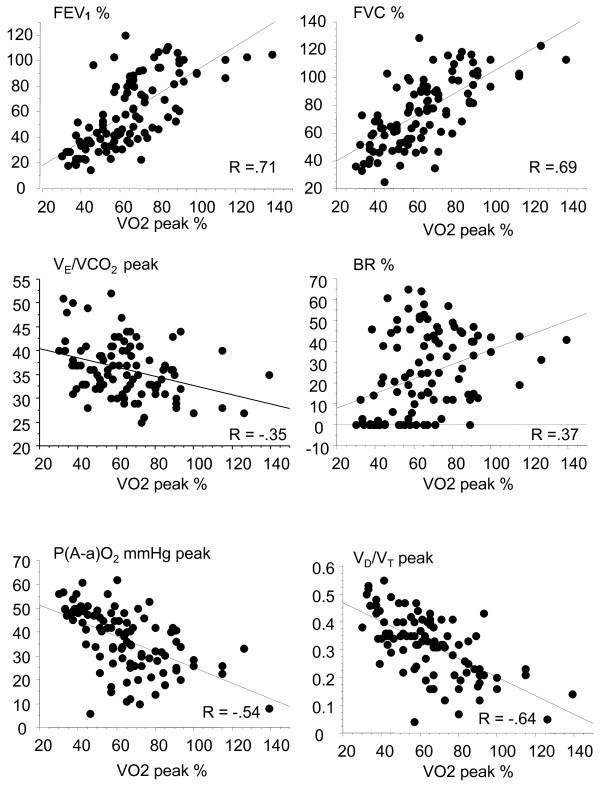
**Correlation between VO**_**2 **_**peak and FEV**_**1**_**, FVC, VE/CO**_**2 **_**peak, BR, P(A-a)O**_**2 **_**peak, and V**_**D**_**/V**_**T **_**peak in CF patients.** VO_2_ peak, FEV_1_, FVC, and BR are expressed as percentage of predicted values. P(A-a)O_2_ peak is expressed as mm Hg.

Significant correlations were observed between VO_2_ peak and nutritional status (BMI, lean body mass), inflammation markers (C-reactive protein [CRP], leukocytosis), resting PFT (FVC, FEV_1_, RV, DLCO, P(A-a)O_2_), and quantifiable parameters of CPET (V_E_ peak, V_E_/VO_2_ peak, V_E_/VCO_2_ peak, BR, V_D_/V_T_ peak, PaO_2_ peak, P(A-a)O_2_ peak, and HR (Table 4 and Figure 1).

**Table 4:** **Correlation of clinical and functional variables with VO**_**2 **_**peak in CF patients**

**Qualitative variables**	**n**		***P *** **value**
Female	102		.22
∆F508 homozygous mutation	79		.02
Exocrine pancreatic insufficiency	102		.55
*Pseudomonas aeruginosa*	99		.36
**Quantitative variables**	**n**	**Correlation (r)**	
Age, years	102	−0.11	.29
BMI, kg/m^2*^	102	0.26	.009
Leukocytosis, 10^9^/mm^3^	70	−0.42	.0003
CRP, mg/L	64	−0.34	.006
Serum albumin, g/L	43	0.34	.02
FEV_1_^a*^	102	0.71	< .0001
FVC^a*^	102	0.69	< .0001
RV^a^	79	−0.58	< .0001
DLCO^a^	68	0.56	< .0001
PaO_2_, mm Hg	98	0.43	< .0001
PaCO_2_, mm Hg	98	0.11	.29
**Cardiopulmonary exercise parameters**	**n**	**Correlation (r)**	
V_E_ peak, L	102	0.64	< .0001
V_E_/VCO_2_ peak*	98	−0.35	< .0001
BR, %*	102	0.37	.0001
V_D_/V_T_ peak*	96	−0.64	< .0001
PaCO_2_ peak, mm Hg*	97	−0.45	< .0001
P(A-a)O_2_ peak, mm Hg*	97	−0.54	< .0001
Lactatemia peak, mmol/L*	86	0.59	< .0001
HR peak^a^	102	0.40	< .0001

^a^Correlations based on percentage of predicted values.

*Selected variables for multivariate stepwise analysis.

The results of the stepwise multiple regression analysis for determinants of exercise capacity are shown in Table 5. Of the variables entered into the model (BMI, FEV_1_, FVC, DLCO, PaO_2_, V_E_/VCO_2_ peak, BR, V_D_/V_T_ peak, PaO_2_ peak, PaCO_2_ peak, P(A-a)O_2_ peak, and lactatemia peak), only FEV_1,_ V_E_/VCO_2_ peak, and BR were found to be independent predictors of exercise capacity (r^2^ = 0.67). Analysis of these three variables showed that, for group 1, 31% of the VO_2_ peak was explained by FEV_1_, whereas the major determinants of the VO_2_ peak in group 2 were BR , FEV_1_ and V_E_/VCO_2_ peak (Table 5).

**Table 5:** **Determinants of VO**_**2 **_**peak in CF patients**

**Variable**	**All patients (n = 102)**	**Group 1 (n = 48)**	**Group 2 (n = 54)**
FEV_1_	50 (0.84 [0.70;0.98])^a^	31 (1.08 [0.70;1.45])^a^	18 (0.67 [0.46;0.88])^a^
BR	12 (−0.62 [−0.82;-0.42])^a^	6 (−0.36 [−0.68;-0.04])^b^	35 (−0.85 [−1.10;-0.59])^a^
V_E_/VCO_2_ peak	5 (−1.13 [−1.56;-0.69])^a^	15 (−0.95 [−1.41;-0.48])^c^	6 (−1.52 [−2.22;-0.82])^a^

Results are expressed as the partial r-square (r^2^), i.e. the percentage of VO_2_ alteration explained by the variable. Coefficient and 95% confidence intervals are shown between parentheses. FEV_1,_ V_E_/VCO_2_ peak, and BR were independent predictors explaining 67% of exercise capacity (r^2^ = 0.67). In group 1, 31% of the VO_2_ peak was explained by FEV_1_, whereas, in group 2, BR was the major determinant explaining 35% of the VO_2_ peak.

Results are derived from multivariable analysis.

^a^Adjusted p value < .0001; ^b^p = .027; ^c^p = .0002.

Separate analysis in the cohort of Lille (75 out of the 102 patients) showed the same results: FEV_1_, BR and V_E_/CO_2_ were independent predictors of exercise capacity (r^2^ = 0.65) (data not shown).

## Discussion

Our study focused on a population of 102 adults with CF who underwent CPET with blood gas analysis at peak exercise. Maximal oxygen uptake was impaired in 82% of patients and was more pronounced in patients with low FEV_1_. We noted a high prevalence of abnormal exercise responses in our population, including abnormal gas exchange, ventilatory and cardiocirculatory responses, and peripheral limitation. The main findings from this study are that exercise intolerance in CF is multifactorial and is correlated mainly with resting pulmonary function, nutritional status, and inflammatory status, but is also affected by the magnitude of the overall ventilatory response during exercise. Multivariate analysis revealed that bronchial obstruction plays a dominant role in patients with severe disease, whereas excessive hyperventilation during exercise was the major determinant of exercise limitation in patients with mild-to-moderate disease.

CF can be associated with abnormal gas exchange, ventilatory, cardiocirculatory, and muscular responses to exercise [3,9,13,29]. In our study, these abnormalities were responsible for limiting the aerobic capacity of 82% of patients, a proportion consistent with previous studies of adult CF patients [5,12,30]. We did not observe a single exercise profile common to all patients, reflecting the complexity of mechanisms involved in exercise limitation in CF patients. Some patients showed abnormalities predominantly in gas exchange, others in the ventilatory response. Still others experienced exercise intolerance despite the absence of ventilatory limitation. The relative contribution of these factors differed between the two groups.

In our study, BMI and CRP levels were strongly correlated with exercise limitation, which is consistent with several studies indicating the importance of inflammatory and nutritional status in exercise limitation. Nutritional status plays a well-established role in CF exercise intolerance [31] and prognosis [32], and may be linked to the chronic inflammation observed in CF patients, which is mainly due to respiratory colonization [33]. Inflammatory markers such as CRP are also negatively associated with exercise capacity in patients with CF [7]. Moreover, inflammation is experimentally correlated with loss of muscle mass [34] and skeletal muscle weakness [10] and could explain the association observed here between CRP, lean body mass, and reduced maximal oxygen uptake.

Multivariate analysis showed that FEV_1_ was the most significant predictor of VO_2_ peak in patients with severe lung disease. This result is consistent with data from earlier studies [3,35] and demonstrates the predominant role of ventilatory disorders in exercise limitation in severe CF patients. Additional functional parameters, such as distension, obstruction, and CO diffusion also correlated with VO_2_ peak, but were not independent predictors. The low BR exhibited by our population is another characteristic of the exercise response in severe CF patients. Tantisira *et al*. showed that the BR index (V_E_/maximal voluntary ventilation calculated at ventilatory threshold) was the most powerful predictor of mortality in CF patients awaiting lung transplantation [36]. This has also been observed in COPD [37] but is not common to all obstructive lung diseases. For example, McNicholl *et al.* reported that only 18% of severe asthma patients had ventilatory limitation due to obstructive lung function [38].

In contrast, the VO_2_ peak was not fully explained by FEV_1_ in patients with mild-to-moderate lung disease, and some patients exhibited impaired aerobic capacity despite having normal resting lung function (Figure 1). Indeed, multivariate analysis showed that two CPET parameters were the major independent determinants of VO_2_ peak in group 2: hyperventilation due to abnormal ventilatory control, resulting in high ventilatory equivalents (as demonstrated by V_E_/VO_2_ and V_E_/VCO_2_ peaks), and BR depletion. Exercise ventilation is regulated by numerous mechanisms, most of which remain incompletely understood [39]. Hyperventilation during exercise reflects a nonspecific response to one or more dysfunctional links in the respiratory chain, but the main cause is not known [40]. In some diseases, such as heart failure, hyperventilation is recognized as a more relevant prognostic factor than VO_2_ peak. The hyperventilatory response may be due to several factors, including inefficient gas exchange as reflected by P(A-a)O_2_ and the V_D_/V_T_ ratio. Although hyperventilation is difficult to relate to other abnormalities, the strong correlation of hyperventilation with oxygen pulse and peak lactatemia suggests that central (cardiovascular) and peripheral (muscle) determinants play a role [10].

In our study, all patients underwent blood gas analysis at peak effort and we noted a high prevalence of gas exchange abnormalities during exercise. It is interesting to note that patients with identical lung function did not all show gas exchange abnormalities. This could be explained by an inadequate ventilatory response in some patients or by a high degree of ventilation-perfusion mismatch. Exercise-induced hypoxemia was common in our study and correlated with VO_2_ peak, workload, peak V_D_/V_T_, and dyspnea assessed by the Borg scale (results not shown). We found that P(A-a)O_2_ correlated well with peak VO_2_, highlighting the relevance of this parameter in gas exchange analysis. Other studies have examined impairment of gas exchange during exercise in CF patients. Nixon *et al.* showed that P_ET_CO_2_ > 41 mm Hg at peak exercise is associated with a twofold higher relative risk of mortality [4]. However, P_ET_CO_2_ is not a reliable marker for PaCO_2_ during exercise and does not allow accurate calculation of dead space [41]. Compared with PFT, CPET with blood gas analysis at peak exercise is better able to assess gas exchange abnormalities and highlight exercise hypoxemia, a recognized prognosis marker, and thus gauge the need for oxygen supplementation.

The primary limitation of our study is its retrospective nature and the possibility of missing data. Peripheral muscle strength was not assessed and might be a significant contributing factor [10]. These results should be confirmed by a prospective study.

## Conclusion

In conclusion, exercise limitation in adult patients with CF correlates with respiratory function as well as nutritional and inflammatory status. This limitation is dependent on FEV_1_ in patients with severe disease but is mainly affected by the magnitude of the ventilatory response to exercise in patients with mild-to-moderate lung disease. CPET thus contributes to a more comprehensive understanding of exercise limitation and can assist in patient management aimed at improving exercise capacity.

## Competing interests

For each author, no significant competing interest exists with any companies or organisations whose products or services are mentioned in this article. The authors declare that they have no competing interests.

## Authors’ contributions

Conception and design: BW, JP and AP; Analysis and interpretation: BW, JP, AP, CT, CL and AD; Drafting the manuscript for important intellectual content: BW, JP, AP, and CL. All authors read and approved the final manuscript.

## Pre-publication history

The pre-publication history for this paper can be accessed here:

http://www.biomedcentral.com/1471-2466/14/74/prepub

## References

[B1] Marcotte JE Grisdale RK Levison H Coates AL Canny GJ Multiple factors limit exercise capacity in cystic fibrosis Pediatr Pulmonol 1986 2 274 281 10.1002/ppul.1950020505 3774384

[B2] Boucher GP Lands LC Hay JA Hornby L Activity levels and the relationship to lung function and nutritional status in children with cystic fibrosis Am J Phys Med Rehabil 1997 76 311 315 10.1097/00002060-199707000-00010 9267191

[B3] Lands LC Heigenhauser GJ Jones NL Analysis of factors limiting maximal exercise performance in cystic fibrosis Clin Sci 1992 83 391 397 1330400 10.1042/cs0830391

[B4] Nixon PA Orenstein DM Kelsey SF Doershuk CF The prognostic value of exercise testing in patients with cystic fibrosis N Engl J Med 1992 327 1785 1788 10.1056/NEJM199212173272504 1435933

[B5] Moorcroft AJ Dodd ME Webb AK Exercise testing and prognosis in adult cystic fibrosis Thorax 1997 52 291 293 10.1136/thx.52.3.291 9093351 PMC1758504

[B6] Pianosi P Leblanc J Almudevar A Peak oxygen uptake and mortality in children with cystic fibrosis Thorax 2005 60 50 54 10.1136/thx.2003.008102 15618583 PMC1747160

[B7] Van de Weert-van Leeuwen PB Slieker MG Hulzebos HJ Kruitwagen CLJJ van der Ent CK Arets HGM Chronic infection and inflammation affect exercise capacity in cystic fibrosis Eur Respir J 2012 39 893 898 10.1183/09031936.00086211 21885387

[B8] Bilton D Dodd ME Abbot JV Webb AK The benefits of exercise combined with physiotherapy in the treatment of adults with cystic fibrosis Respir Med 1992 86 507 511 10.1016/S0954-6111(96)80012-6 1470709

[B9] Almajed A Lands LC The evolution of exercise capacity and its limiting factors in Cystic Fibrosis Paediatr Respir Rev 2012 13 195 199 10.1016/j.prrv.2012.01.001 23069115

[B10] Troosters T Langer D Vrijsen B Segers J Wouters K Janssens W Gosselink R Decramer M Dupont L Skeletal muscle weakness, exercise tolerance and physical activity in adults with cystic fibrosis Eur Respir J 2009 33 99 106 10.1183/09031936.00091607 18715878

[B11] Selvadurai HC Allen J Sachinwalla T Macauley J Blimkie CJ Van Asperen PP Muscle function and resting energy expenditure in female athletes with cystic fibrosis Am J Respir Crit Care Med 2003 168 1476 1480 10.1164/rccm.200303-363OC 14500260

[B12] Cerny FJ Pullano TP Cropp GJ Cardiorespiratory adaptations to exercise in cystic fibrosis Am Rev Respir Dis 1982 126 217 220 7103246 10.1164/arrd.1982.126.2.217

[B13] Shah AR Gozal D Keens TG Determinants of aerobic and anaerobic exercise performance in cystic fibrosis Am J Respir Crit Care Med 1998 157 1145 1150 10.1164/ajrccm.157.4.9705023 9563732

[B14] Lebecque P Lapierre JG Lamarre A Coates AL Diffusion capacity and oxygen desaturation effects on exercise in patients with cystic fibrosis Chest 1987 91 693 697 10.1378/chest.91.5.693 3568772

[B15] Bradley S Solin P Wilson J Johns D Walters EH Naughton MT Hypoxemia and hypercapnia during exercise and sleep in patients with cystic fibrosis Chest 1999 116 647 654 10.1378/chest.116.3.647 10492266

[B16] McKone EF Barry SC Fitzgerald MX Gallagher CG Role of arterial hypoxemia and pulmonary mechanics in exercise limitation in adults with cystic fibrosis J Appl Physiol 2005 99 1012 1018 10.1152/japplphysiol.00475.2004 15860682

[B17] Marcus CL Bader D Stabile MW Wang CI Osher AB Keens TG Supplemental oxygen and exercise performance in patients with cystic fibrosis with severe pulmonary disease Chest 1992 101 52 57 10.1378/chest.101.1.52 1729110

[B18] ATS/ACCP Statement on cardiopulmonary exercise testing Am J Respir Crit Care Med 2003 167 211 277 12524257 10.1164/rccm.167.2.211

[B19] Stevens D Oades PJ Armstrong N Williams CA A survey of exercise testing and training in UK cystic fibrosis clinics J Cyst Fibros 2010 9 302 306 10.1016/j.jcf.2010.03.004 20359963

[B20] Miller MR Hankinson J Brusasco V Burgos F Casaburi R Coates A Crapo R Enright P van der Grinten CPM Gustafsson P Jensen R Johnson DC MacIntyre N McKay R Navajas D Pedersen OF Pellegrino R Viegi G Wanger J Standardisation of spirometry Eur Respir J 2005 26 319 338 10.1183/09031936.05.00034805 16055882

[B21] Macintyre N Crapo RO Viegi G Johnson DC van der Grinten CPM Brusasco V Burgos F Casaburi R Coates A Enright P Gustafsson P Hankinson J Jensen R McKay R Miller MR Navajas D Pedersen OF Pellegrino R Wanger J Standardisation of the single-breath determination of carbon monoxide uptake in the lung Eur Respir J 2005 26 720 735 10.1183/09031936.05.00034905 16204605

[B22] Wanger J Clausen JL Coates A Pedersen OF Brusasco V Burgos F Casaburi R Crapo R Enright P van der Grinten CPM Gustafsson P Hankinson J Jensen R Johnson D Macintyre N McKay R Miller MR Navajas D Pellegrino R Viegi G Standardisation of the measurement of lung volumes Eur Respir J 2005 26 511 522 10.1183/09031936.05.00035005 16135736

[B23] Aguilaniu B Richard R Costes F Bart F Martinat Y Stach B Aguilaniu B Richard R Costes F Bart F Martinat Y Stach B Denjean A Scientific Council of the French Lung Society [Cardiopulmonary exercise testing] Rev Mal Respir 2007 24 2S111 2S160 17389842

[B24] Wallaert B Talleu C Wemeau-Stervinou L Duhamel A Robin S Aguilaniu B Reduction of maximal oxygen uptake in sarcoidosis: relationship with disease severity Respiration 2011 82 501 508 10.1159/000330050 21934275

[B25] Sorbini CA Grassi V Solinas E Muiesan G Arterial oxygen tension in relation to age in healthy subjects Respiration 1968 25 3 13 10.1159/000192549 5644025

[B26] Hansen JE Sue DY Wasserman K Predicted values for clinical exercise testing Am Rev Respir Dis 1984 129 S49 S55 6421218 10.1164/arrd.1984.129.2P2.S49

[B27] Sauerbrei W The Use of Resampling Methods to Simplify Regression Models in Medical Statistics Journal of the Royal Statistical Society: Series C (Applied Statistics) 1999 48 313 329 10.1111/1467-9876.00155

[B28] Vaincre La Mucoviscidose http://www.vaincrelamuco.org/. 2011 Rapport annuel. 2012

[B29] Leroy S Perez T Neviere R Aguilaniu B Wallaert B Determinants of dyspnea and alveolar hypoventilation during exercise in cystic fibrosis: impact of inspiratory muscle endurance J Cyst Fibros 2011 10 159 165 10.1016/j.jcf.2010.12.006 21345745

[B30] Godfrey S Mearns M Pulmonary function and response to exercise in cystic fibrosis Arch Dis Child 1971 46 144 151 10.1136/adc.46.246.144 5576246 PMC1647451

[B31] Gulmans VA de Meer K Brackel HJ Helders PJ Maximal work capacity in relation to nutritional status in children with cystic fibrosis Eur Respir J 1997 10 2014 2017 10.1183/09031936.97.10092014 9311494

[B32] Nguyen S Leroy S Cracowski C Perez T Valette M Neviere R Aguilaniu B Wallaert B Prognostic value of clinical exercise testing in adult patients with cystic fibrosis Rev Mal Respir 2010 27 219 225 10.1016/j.rmr.2010.01.009 20359613

[B33] Van de Weert-van Leeuwen PB Arets HGM van der Ent CK Beekman JM Infection, inflammation and exercise in cystic fibrosis Respir Res 2013 14 32 10.1186/1465-9921-14-32 23497303 PMC3599254

[B34] Van Heeckeren AM Tscheikuna J Walenga RW Konstan MW Davis PB Erokwu B Haxhiu MA Ferkol TW Effect of Pseudomonas infection on weight loss, lung mechanics, and cytokines in mice Am J Respir Crit Care Med 2000 161 271 279 10.1164/ajrccm.161.1.9903019 10619831

[B35] Klijn PHC van der Net J Kimpen JL Helders PJM van der Ent CK Longitudinal determinants of peak aerobic performance in children with cystic fibrosis Chest 2003 124 2215 2219 10.1378/chest.124.6.2215 14665503

[B36] Tantisira KG Systrom DM Ginns LC An elevated breathing reserve index at the lactate threshold is a predictor of mortality in patients with cystic fibrosis awaiting lung transplantation Am J Respir Crit Care Med 2002 165 1629 1633 10.1164/rccm.2105090 12070064

[B37] Medoff BD Oelberg DA Kanarek DJ Systrom DM Breathing reserve at the lactate threshold to differentiate a pulmonary mechanical from cardiovascular limit to exercise Chest 1998 113 913 918 10.1378/chest.113.4.913 9554625

[B38] McNicholl DM Megarry J McGarvey LP Riley MS Heaney LG The utility of cardiopulmonary exercise testing in difficult asthma Chest 2011 139 1117 1123 10.1378/chest.10-2321 21292756

[B39] Dempsey JA Challenges for future research in exercise physiology as applied to the respiratory system Exerc Sport Sci Rev 2006 34 92 98 10.1249/00003677-200607000-00002 16829735

[B40] Péronnet F Aguilaniu B Lactic acid buffering, nonmetabolic CO2 and exercise hyperventilation: a critical reappraisal Respir Physiol Neurobiol 2006 150 4 18 10.1016/j.resp.2005.04.005 15890562

[B41] Lewis DA Sietsema KE Casaburi R Sue DY Inaccuracy of noninvasive estimates of VD/VT in clinical exercise testing Chest 1994 106 1476 1480 10.1378/chest.106.5.1476 7956406

